# PROTOCOL: A comparison of the effectiveness of cognitive behavioural interventions based on delivery features for elevated symptoms of depression in adolescents

**DOI:** 10.1002/cl2.1073

**Published:** 2020-01-07

**Authors:** Gretchen J. Bjornstad, Shreya Sonthalia, Benjamin Rouse, Luke Timmons, Laura Whybra, Nick Axford

**Affiliations:** ^1^ Dartington Service Design Lab Buckfastleigh UK; ^2^ University of Exeter Medical School University of Exeter Exeter UK; ^3^ Center for Clinical Evidence and Guidelines ECRI Institute Plymouth Meeting Pennsylvania; ^4^ Stand Together Inc. Arlington Virginia; ^5^ Peninsula Medical School Faculty of Health: Medicine, Dentistry and Human Sciences Plymouth University Plymouth UK; ^6^ National Institute for Health Research (NIHR) Applied Research Collaboration (ARC) South West Peninsula Plymouth UK

## Abstract

This is the protocol for a Campbell review. The primary aim is to estimate the relative efficacy of different modes of CBT delivery compared with control conditions for reducing depressive symptoms in adolescents. The secondary aim is to compare the different modes of delivery with regards to intervention completion/attrition (used as a proxy for intervention acceptability). The review will provide relative effect estimates and ranking probabilities for each outcome based on intervention delivery.

## 1 BACKGROUND

### 1.1 Description of the condition

Depression is a public health problem and common among adolescents. It is estimated that around one in ten adolescents in the USA experience at least one major depressive episode per year (Center for Behavioral Health Statistics and Quality, 2015). In Europe, the prevalence of depression in adolescents has been reported using baseline data from a randomised controlled trial (RCT), which found the prevalence ranging from 7.1–19.4% across 11 countries (Balázs et al., 2012).

An analysis in the United States found the lifetime prevalence of major depressive disorder to be 16.6%, with a median age of onset of 32 years (Kessler et al., 2005). In terms of prevalence in children and young people, an analysis of 26 epidemiological studies of children and adolescents born between 1965 and 1996 found that rates of depression (any depression, major depressive disorder, or major depressive episode), established through diagnostic interview, have not increased over a 30‐year period (Costello, Erkanli, & Angold, 2006). This study found that the prevalence of depression in adolescents was 5.7%.

A diagnosis of major depressive disorder, according to the DSM‐V criteria, is characterised by the presence of five or more symptoms, such as a persistent depressed mood, a loss of interest or pleasure in daily activities, sleep problems, change in appetite or weight, fatigue, feelings of worthlessness or guilt, diminished concentration and suicidal thoughts, consistently for at least a 2‐week period (American Psychiatric Association 2013). Clinically significant distress or impairment in social, occupational, or other important areas of functioning must also be present.

The incidence of major depressive disorder in children and adolescents is associated with lifetime psychiatric comorbidity, risk of suicidality, functional impairments and recurrence (Rohde, Lewinsohn, Klein, Seeley, & Gau, 2013). An analysis of global data found that neuropsychiatric disorders were the main cause of disease burden for young people aged 10 to 24 years, with most of these accounted for by unipolar depressive disorders (Gore et al., 2011).

In addition, a history of depressive episodes or elevated symptoms of depression are significant risk indicators of later major depressive disorder and can have a negative impact on the quality of life (Bertha & Balázs, 2013). An analysis from the Christchurch Health and Development Study birth cohort in New Zealand found that subthreshold depression in young people aged 17 to 18 was associated with later depression and suicidal tendencies up to age 25 (Fergusson, Horwood, Ridder, & Beautrais, 2005). However, a prospective longitudinal cohort study in Australia found that although the diagnosis of depression in adolescence predicts diagnosis in young adulthood, the rates of disorder dropped by the late 20s (Patton et al., 2014). Remission was most likely in cases where adolescent depression was brief in duration.

The evidence for the incidence, impact and prognosis of adolescent depression continue to indicate that it is important to identify the most effective interventions to reduce depressive symptoms and limit the duration of diagnoses to improve quality of life in adolescence and into adulthood.

### 1.2 Description of the intervention

The focus of this review will be on interventions that are based on cognitive behavioural therapy (CBT) and delivered through various modalities. CBT is widely used to treat depression among children, adolescents and adults and is one of several interventions recommended for treating depression in children and adolescents by the National Institute for Health and Care Excellence in the UK and the American Academy of Child and Adolescent Psychiatry (Birmaher & Brent, 2007; NICE 2017).

CBT is a psychotherapy based on the premise that cognitions, behaviour patterns and emotions are linked, and that cognitive and behavioural techniques can produce changes in these links (Kendall & Panichelli‐Mindel, 1995). According to this model, adolescents with depressive symptomology have negative perceptions about themselves, the world and the future, which affect behaviours and sustain the feelings of low self‐esteem and hopelessness (Dobson & Dozois, 2001). For example, depressed individuals will be selective in choosing the evidence for their performance, such that only those instances that support poor performance are remembered, which leads to behaviours that contribute to the development and persistence of depression, such as reduced engagement in activities. This reduced engagement reduces the chance of positive reinforcement. A reduction in positive reinforcement for healthy behaviours may lead to depressive symptomology, and depressed individuals often give up activities that they value. Thus, CBT aims to modify the relationship between thoughts, behaviours and emotions.

### 1.3 How the intervention might work

This review is concerned with examining various delivery modalities of CBT. CBT can be delivered by a therapist in groups or individually, or through self‐help. Therapist support may be delivered face‐to‐face or remotely, which can be real‐time or delayed. Furthermore, CBT can be delivered via telephone calls or text messages with a therapist, or via the web through web‐based programmes that may or may not include communication with a therapist (Rathbone, 2017). Potential reasons for virtual appointments are to improve accessibility to therapy and reduce costs. The dose (number, duration and frequency of sessions) of CBT varies and the dose‐response relationship is not well understood (Girlanda et al., 2016).

The content and dose are often similar between face‐to‐face and remote therapy. Self‐help is a mode of delivery that is independent of professional contact and can be delivered via books, computer programmes, or other media. Therapist support can be provided alongside self‐help to guide the patient through the intervention (Cuijpers, Donker, van Straten, Li, & Andersson, 2010). Self‐help CBT can be standardised (i.e., the material is not tailored to individuals and is the same package for all) or personalised (i.e., the material is tailored to individual needs), and it may or may not be interactive.

### 1.4 Why it is important to do this review

Existing research does not provide clear conclusions regarding the relative effectiveness of the different delivery modalities of CBT for depression in children and adolescents.

A network meta‐analysis of psychotherapies for depression in children and adolescents found that only interpersonal therapy and CBT were significantly more effective than control conditions and were more effective in reducing depressive symptoms than alternative psychotherapies such as play therapies and problem‐solving therapy (Zhou et al., 2015). However, the review included different delivery modalities of each psychotherapy in the same node and therefore could not draw conclusions about the relative effectiveness of these modalities.

A recent systematic review looked at the effectiveness of computerised therapies for anxiety and depression in children and young people (Pennant et al., 2015). It identified studies testing three programmes for depression and two programmes aimed at both anxiety and depression; these included interactive games and standardised educational programmes. All of the programmes for depression were rated by the authors as having low therapist input. One programme for depression and anxiety in the general population was rated as having low therapist input, but the remaining two programmes were for populations at risk of anxiety and depression and involved some therapist input. The review found that computerised CBT was more effective than nontherapeutic controls, but that face‐to‐face therapy was more effective than computerised CBT. A limitation of the review is that it looked at computerised therapies as a whole, rather than categorising them according to whether they were solely self‐help interventions or included therapist support. Similarly, a review of online and social networking interventions for depression in young people found that online interventions with a cognitive behavioural focus were promising in terms of reducing depression (Rice et al., 2014). This review included studies with varying levels of support, usually from moderators or tutors. It also found a lot of variation between interventions in terms of dropout rates and it was not clear whether the level of support was related to attrition.

Another review that found computerised CBT interventions to be effective in reducing depressive symptoms in children and young people up to age 25 did not differentiate between therapist‐guided and unguided self‐help formats of CBT interventions (Ebert et al., 2015). Similarly, a review of computerised CBT for anxiety and depression found that included studies varied considerably in terms of therapist support (Richardson, Stallard, & Velleman, 2010). This is potentially important because there is some evidence based on an analysis of computerised psychotherapies with adults that the effect on depressive symptoms is moderated by the level of therapist support, with larger effects associated with therapist involvement (Andersson & Cuijpers, 2009). A survey of young people using Child and Adolescent Mental Health Services in the UK also found that most young people would prefer to talk to a therapist, with only 9% preferring to use a computer programme on their own (Stallard, Velleman, & Richardson, 2010).

Existing reviews are limited by the lack of primary research comparing the effectiveness of multiple modes of delivering CBT directly, with most studies comparing computerised forms of CBT that are either purely self‐help or self‐help with therapist support with waitlist, no treatment, or treatment as usual controls (Calear & Christensen, 2010; Fleming et al., 2014). While there are studies that compare the effectiveness of a particular mode of delivery of CBT to no intervention (e.g., van der Zanden, Kramer, Gerrits, & Cuijpers, 2012) or another non‐CBT control condition (e.g., Reynolds & Coats, 1986), few studies conduct a head‐to‐head evaluation of two different modes of delivering CBT.

These existing reviews (Calear & Christensen, 2010; Ebert et al., 2015; Fleming et al., 2014; Pennant et al., 2015; Rice et al., 2014) all combine self‐help with therapist support and self‐help without therapist report, making it impossible to determine the relative effectiveness of these two delivery modes and leaving open the questions as to whether the addition of therapist support leads to greater effectiveness or patient engagement.

To address this gap, this review will conduct a network meta‐analysis, a method that includes direct and indirect evidence of the relative effectiveness of different interventions and thus allows comparison of pairs of interventions where there are few or no studies that have tested the two interventions in a head‐to‐head trial. This method will also allow for examination of the ranking probabilities of competing modes of delivering CBT based on their relative effectiveness for reducing depression among adolescents (Salanti, Ades, & Ionnidis, 2011).

The findings of this review will have implications for policy and practice and the future funding of mental health service provision by providing an understanding of how different CBT delivery modes compare with one another on a subpopulation of adolescents with elevated symptoms of depression. As delivery modalities will differ in terms of demands on resources, this review may have important cost‐benefit implications, which could be examined in further research (Arnberg, Linton, Hultcrantz, Heintz, & Jonsson, 2014).

## 2 OBJECTIVES

The current review aims to estimate the relative efficacy of different modes of CBT delivery compared with control conditions for reducing depressive symptoms in adolescents. The review will provide relative effect estimates and ranking probabilities on the effectiveness of interventions to reduce depressive symptoms in adolescents based on intervention delivery.

Primary question

1. In terms of reducing depressive symptoms in adolescents with elevated symptoms of depression, how do cognitive behavioural interventions differentiated by delivery modes compare to one another and to control groups?
  Secondary question
2. With regards to intervention completion/attrition (used as a proxy for intervention acceptability), how do cognitive behavioural interventions (for depressive symptoms in adolescents with elevated symptoms of depression) differentiated by delivery modes compare to one another and to control groups?

## 3 METHODS

### 3.1 Criteria for considering studies for this review

#### 3.1.1 Types of studies

This review will look exclusively at RCTs with pre‐ and postdata, including cluster RCTs (i.e., where groups of participants, such as a classroom, rather than individuals, are the unit of random allocation). Quasi‐randomised trials (i.e., use of quasi‐random methods of allocation such as alternation, date of birth, case record number) and controlled clinical trials will be ineligible in order to minimise bias that could threaten the validity of the network meta‐analysis. Cross‐over studies (i.e., where study groups receive two or more interventions in different sequences) will be included only if they are RCTs and if they provide data at the end of the first stage. Multiarm trials will be included.

#### 3.1.2 Types of participants

The population of interest is adolescents with elevated, clinically relevant symptoms of depression as measured by validated self‐reported measures or diagnostic instruments. We will include adolescents who meet diagnostic criteria for major depressive disorder.

### 3.2 Age

All studies conducted with adolescents aged 10 to 19 years will be included, in line with the WHO definition of adolescence. Studies conducted with secondary, middle or high school students will also be included (where the age range may differ slightly). Where studies include younger children or adult populations along with adolescents, they will be included only if the data on adolescents are reported separately.

### 3.3 Specific characteristics

Studies that include participants of a specific characteristic (e.g., participants of a particular ethnicity or those in families where parents have divorced) will not be excluded unless the intervention has been designed specifically for the population or has made adaptations to the content of the intervention and a threat to the transitivity assumption is therefore present.

### 3.4 Diagnosis

This review will focus on adolescents with elevated symptoms of depression, that is, clinically relevant symptoms of depression that may or may not meet diagnostic criteria for major depressive disorder. Elevated symptoms of depression may be established using diagnostic instruments or scores on self‐report measures.

The diagnostic instruments that will be considered are as follows.

- The Schedule for Affective Disorders and Schizophrenia for School‐age Children.
- The Diagnostic Interview Schedule for Children.
- The Diagnostic Interview for Children and Adolescents‐Revised.
- The Child and Adolescent Psychiatric Assessment.

We will include studies with participants scoring in the clinical range of symptoms of depression based on self‐reported measures.

Table 1 lists score cut points for some common self‐reported measures that will be included. The eligibility criteria will be considered first, followed by the baseline scores to identify if the mean score is above the cut point. If other measures are used, they will be considered for inclusion based on their validity as measures of depression in adolescents.

In cases where the information provided in the study is unclear and the author does not provide further clarification, we will include studies if participants are included based on depressive symptoms.

Studies using measures that have not been validated as a depression measure for children and adolescents against a diagnostic tool, which have a low reliability (e.g., the Bellevue Index of Depression—low reliability reported in Kazdin, French, Unis, & Esveldt‐dawson, 1983), or where the method to establish elevated symptoms of depression is unclear will be excluded in a sensitivity analysis.

Other mental health constructs such as “attributional style” are not considered as elevated depressive symptoms and thus will be excluded. Studies that include adolescents who are deemed to be at risk of developing any form of depressive disorder but who do not display elevated symptoms will be excluded. The exception to this is when the mean depression score at baseline for the intervention and comparison groups falls in the elevated level of depressive symptoms mentioned above.

Studies with adolescents with any comorbid disorders (e.g., depression and anxiety, depression and schizophrenia) will only be included if the focus of the intervention is the treatment of depression, not comorbid conditions.

Studies will also be excluded if their inclusion criteria include adolescents with cognitive impairments (e.g., learning difficulties and autism), or adolescents with chronic or acute physical health conditions, or if the reports state that adolescents with these types of impairments or conditions were part of the study sample. The purpose of this last criterion is to limit the variation in populations within and across studies in the network, as it is an important effect modifier that has implications for the validity of the network meta‐analysis.

#### 3.4.1 Types of interventions

The review will include cognitive behavioural interventions that aim to reduce symptoms of depression in adolescence, irrespective of delivery mode. For the purposes of this review, an intervention will be considered a cognitive behavioural intervention if it includes (a) evaluation of cognition to identify dysfunctional cognition, (b) cognitive restructuring to adopt helpful cognition and (c) a component focusing on behaviour: behavioural activation, problem solving, social skills training or relaxation techniques. We recognise that variation in the third component may confound the estimated difference between treatment delivery modes, and if we are able to identify these types of differences between the interventions, we will test this using sensitivity analyses. Similarly, if the description of the intervention is not clear but is described as having a more cognitive or behavioural focus but is likely to be CBT, we will include the relevant studies and test in sensitivity analyses as these interventions may be partial CBT with more cognitive or more behavioural foci (Hetrick et al., 2015).

If the description of the intervention in source documents is not adequate to make an assessment on inclusion based on the above criteria, the author(s) will be contacted. If we do not receive further details from the author(s), we will include studies that identify the intervention as CBT and exclude studies that do not identify the intervention as CBT.

Studies evaluating interventions that do not have all three CBT components listed above, or which are not identified by the authors as CBT, will be excluded.

In line with the above conceptualisation of CBT, interventions such as acceptance and commitment therapy, mindfulness‐based cognitive therapy and dialectical behaviour therapy that are rooted in principles different from those of CBT and focus on helping people to accept thoughts in a nonjudgemental manner will be excluded (e.g., Hayes, Strosahl, & Wilson, 1999; Linehan et al., 2006; Segal, Williams, & Teasdale, 2012).

Interventions will be placed according to their mode of delivery into the following five categories.

1. Therapist‐delivered CBT in one‐to‐one sessions: This includes CBT delivered by a therapist to individual clients either in face‐to‐face sessions or remotely but in real‐time (for example, audio or video call, live messaging).
2. Therapist‐delivered CBT in group sessions: This is similar to the above, but sessions are conducted for a group of clients rather than an individual client.
3. Therapist‐led CBT delivered remotely: This includes CBT that is delivered by a therapist remotely—for example, emails, Skype and text messaging. The delivery can be to individual clients or groups of clients. If an intervention combines real‐time and delayed support, it will be categorised based on the primary intended means of therapist contact.
4. Unguided self‐help: This involves educating the client in the principles of CBT through reading material and helping them apply it through quizzes and activities. Traditionally, this was referred to as bibliotherapy and included workbooks. CBT can now be provided through various technological platforms (such as smartphone applications or browser‐based programmes), and include audio files and videos in addition to text. When delivered electronically, self‐help may include additional features such as reminders and some basic guidance on how to use the materials.
5. Self‐help with therapist support: This involves material to introduce and guide the client through CBT, alongside support from a therapist. For example, clients might gain an understanding of the approach to thoughts via the workbook and could be given homework and have regular feedback calls with a therapist.

Comparisons will be classified as (a) no intervention, (b) treatment as usual and (c) placebo. In order to be included in the current review, studies must do one of the following.

1. Compare two cognitive behavioural interventions delivered through different delivery modes. Studies comparing two versions of cognitive behavioural interventions that have the same delivery mode will not be included in the network meta‐analyses. If such a study also has another relevant intervention or control group, the two different groups with a common delivery mode will be considered as one intervention for the analysis. The way in which the common effect size will be determined is explained below.
2. Compare a cognitive behavioural intervention with a no intervention, placebo, services as usual control group. Comparisons in which any pharmacological treatment (e.g., antidepressants), complementary and alternative medicine (e.g., light therapy, acupuncture) or physical interventions (e.g., yoga, exercise) are explicitly provided (i.e., not as services as usual) will not be considered because they are beyond the scope of this review. Waitlist controls will be classified as no intervention controls. Services‐as‐usual will be grouped together to avoid disconnecting the network. Psychological placebos may include psychoeducation or attention placebos that are not expected to have any impact on the outcome of interest. Psychoeducation is the provision of information about a mental health condition without the provision of therapy. Attention placebo conditions provide similar time and attention from a therapist to participants without the provision of the active therapeutic intervention. Where services‐as‐usual or a placebo are not adequately described in source documents, the author(s) will be contacted. If sufficient detail is not obtained, the study in question will be excluded.

Studies where CBT is implemented in combination with another intervention will be excluded unless the comparison group also received the additional intervention, meaning that the effects of the other intervention would be controlled for.

A sensitivity analysis separating therapist‐led placebos and self‐help placebos will be carried out if both types of placebos are present in the included studies.

We assume that any adolescent who meets the inclusion criteria is, in principle, equally likely to be randomised to any of the eligible interventions.

**Figure 1:**
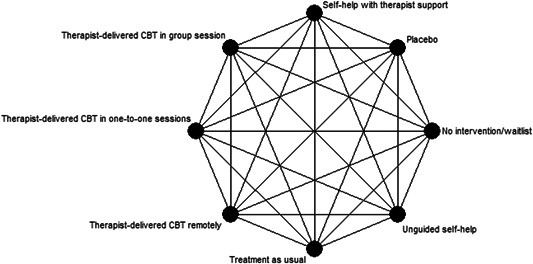
Draft network including all treatment and control nodes. CBT, cognitive behavioural therapy

Figure 1 shows all possible intervention and control comparisons.

#### 3.4.2 Types of outcome measures

##### Primary outcomes

The primary outcome for the review will be depressive symptom final score at postintervention and at 6 to 12 month follow‐up assessments. To be included in the analysis, assessment of depressive symptoms must be self‐report using a validated measure, such as most of those listed in Table 1 (the CDRS‐R and the HDRS will not be included as outcome measures as they are not self‐report measures). We will consider other measures of depression symptoms for inclusion in the analysis if used by studies and validated. In cases where there are multiple measurement points within the 6 to 12 month timeframe, we will use the measurement point closest to 12 months.

Where insufficient information is provided for endpoint values, we will contact the author(s) for the required data. If those data cannot be provided, we will describe the results in the narrative summary.

Only continuous measures of depression symptoms assessed using a validated assessment or diagnostic tool will be considered. In cases where a study uses multiple appropriate measures for depressive symptoms, we will prioritise the measure that is most used across all included studies for consistency across the network. In cases where one particular measure used in a study is not more common in the network than another, we will use the measure that the authors of the study consider to be the primary measure.

##### Secondary outcomes

The secondary outcome is acceptability of the intervention. This will be defined as completing the intervention. This will be operationalised as intervention discontinuation or attrition or the number of participants who dropped out of the study by posttest as a dichotomous outcome (Kaltenthaler et al., 2008).

##### Search methods for identification of studies

###### Electronic searches

A search of the following electronic databases will be conducted by the Cochrane Common Mental Disorders Group Trials Search Coordinator.

- PsycINFO
- EMBASE
- PubMED (overlaps with MEDLINE)
- International Bibliography of Social Science (IBSS)
- Cumulative Index to Nursing and Allied Health Literature (CINAHL)
- Health Technology Assessment (HTA) Database
- The Cochrane Central Register of Controlled Trials (CENTRAL)

PsychINFO and Embase will be searched through OVID and PubMED through NCBI. IBSS will be searched using the ProQuest platform, while CINAHL will be via EBSCOhost. HTA is hosted by the Centre for Reviews and Dissemination at the University of York.

The following trial registries will be searched.

- ClinicalTrials.gov
- International Clinical Trials Registry Platform (WHO)

Search strategies will be tailored for each of the databases. The main search strategy will be as follows.

INTERVENTION: [(cognitive OR behavio(u)r*) AND (therapy OR bibliotherapy OR intervention OR program* OR prevention OR treatment OR self‐help OR psychoeducation OR modification OR training)] or CBT

POPULATION: [(child* OR adolescen* OR preadolescen* OR minor* OR young* OR youth OR student* OR teen* OR girl* OR boy* OR male* OR female* OR school OR juvenile*) AND (depression OR depressive OR depressed OR low mood OR mood disorder OR dysthymi*)]

STUDY DESIGN: random* OR control OR experiment* OR clinical study OR trial OR RCT

##### Searching other resources

The following sources will be hand‐searched (applying appropriate filters).

- Headspace (Australian National Youth Mental Health Foundation) Research Database—the research database for an evidence map of published systematic reviews and controlled studies on depression interventions for young people (Callahan, Liu, Purcell, Parker, & Hetrick, 2012). The database will be filtered for depression, CBT and trials and then hand‐searched.
- www.evidencebasedpsychotherapies.org—a database on RCTs of psychotherapies; this will be filtered by depression and then hand‐searched.

We will search the reference lists of the following recent reviews on psychotherapies for depression in children and young people: Calear and Christensen 2010, Ebert et al., 2015, Fleming et al., 2014, Pennant et al., 2015, Rice et al., 2014 and Zhou et al., 2015, as well as reviews retrieved in the search. We will also use Google Scholar with appropriate search terms from the search strategy to identify newer studies that have cited these reviews.

References of all included studies will be checked to identify further studies.

Grey literature will be identified through handsearching and review of reference lists as described above and by contacting authors of these reviews and included studies. Additional methods to identify grey literature will include searching ProQuest Dissertations and Theses (Global and UK & Ireland), Index to Theses in Great Britain and Ireland, Educational Technology and E‐Learning (EdITLib), PsycEXTRA, Open Grey and Google (using advanced search).

Any studies identified after completion of analysis will be included in the review as “studies awaiting classification”. References will be managed using Endnote X7 and Mendeley.

#### 3.4.3 Data collection and analysis

##### Selection of studies

We will use Cochrane's Screen4Me workflow to help assess the search results. Screen4Me comprises three components: known assessments—a service that matches records in the search results to records that have already been screened in Cochrane Crowd and been labelled as an *RCT* or as *Not an RCT*; the RCT classifier—a machine learning model that distinguishes RCTs from non‐RCTs; and if appropriate, Cochrane Crowd (http://crowd.cochrane.org)—Cochrane's citizen science platform where the Crowd help to identify and describe health evidence.

For more information about Screen4Me and the evaluations that have been done, please go to the Screen4Me webpage on the Cochrane Information Specialist's portal: https://community.cochrane.org/organizational‐info/resources/resources‐groups/information‐specialists‐portal/searching‐conducting. In addition, more detailed information regarding evaluations of the Screen4Me components, can be found in the following publications: Marshall, Noel‐Storr, Kuiper, Thomas, & Wallace, 2018, McDonald, Noel‐Storr, & Thomas, 2017, Noel‐Storr and the Project Transform team, 2018, Thomas et al., 2017.

Unique references will be exported to Mendeley and sent through the Screen4Me workflow. The remaining references will then be screened for relevance by title and abstract by two of four independent authors (G. B., L.W., S. S. and B. R.). Screening and data extraction will be managed and stored using Covidence.

The full text of potentially relevant articles will also be screened independently by two of four authors (G. B., L. W., S. S. and BR) for inclusion. Any discrepancy will be resolved by consensus and discussion with the principal investigator. The screening checklist will also be reviewed by the second author. Eligibility will be assessed using a predesigned form based on the inclusion criteria. Articles excluded at this stage will be reported in a table with reasons for exclusion. We will report interrater reliability for study identification. The screening process will be reported using a PRISMA flow chart.

The screening checklist will include the following.

1. Does the study include a relevant intervention?
  a. Is the intervention based on CBT?
  b. Does the intervention target depression?
2. Is the study conducted with adolescents, or students in secondary, middle or high school, or is the mean age between 10 and 19 years?
  a. Specify mean age.

3. Is the study conducted with participants who have elevated levels of depression?
  a. Specify screening measure and cutoff score.

4. Is the study an RCT with two different nodes in the network?
  a. Confirm if RCT.
  b. Specify all groups and potential nodes.

### 3.5 Description of methods used in primary research

The review will only include RCTs. The most common comparison groups are expected to be no intervention and services‐as‐usual. Most studies will recruit and randomise individuals based on an assessment of depressive symptoms. Some studies might randomise clusters (such as classrooms). Studies may use any of a number of measures of depression, with assessments taking place before the intervention (as screening and baseline assessment) and immediately after the intervention. Some studies may include one or more subsequent follow‐ups. While most studies are likely to use the same measures before and after the intervention, some studies may use different measures. Similarly, while most studies are likely to use one depression scale, studies may employ more than one scale.

### 3.6 Criteria for determination of independent findings

Multiple publications of the same study will be examined as a single study.

### 3.7 Studies with two or more groups

In a multiarm trial where more than one mode of delivering CBT is evaluated, we will keep the groups separate and account for correlations due to multiarm trials as recommended by Salanti, Higgins, Ades, & Ioannidis, 2008.

For multiarm trials where not all arms are relevant, we will not include nonrelevant arms in the analysis but will include them in the “Table of Characteristics”.

#### 3.7.1 Data extraction and management

Data extraction will be done initially (first 10 studies) by four authors (G. B., L. W., S. S. and B. R.) to ensure consistency. Subsequent data extraction will be done by one author and then checked by another author to identify and resolve potential discrepancies, with discussion with a third author if required. Study coding for network node will be conducted by one author, with a proportion conducted by two authors, with discrepancies being resolved by a third author. We will report interrater reliability for node identification for those that are coded by two authors. A second author will also check through all network node coding for reasoning.

Studies will be coded for study design, characteristics of participants and intervention. The study design will include a number of groups, sample size, attrition, recruitment and referral procedures, unit and method of randomisation, data collection methods and timing. Participant characteristics will include age, gender, ethnicity, socioeconomic status, baseline depressive symptoms and eligibility criteria. Intervention characteristics will include content, format, delivery modality including details on provider (e.g., therapist qualifications and training, technology platform), customisation, setting, dosage and implementation fidelity. These details will be coded for all intervention and control groups. Based on the data extraction, each group will be classified as one of the network nodes.

Data extraction will be undertaken using a standard extraction form, which will be pretested.

#### 3.7.2 Assessment of risk of bias in included studies

Four review authors (G. B., L. W., S. S. and B. R.) will independently assess the risk of bias using the Cochrane Risk of Bias Tool (Higgins, Deeks, & Altman, 2011). The following domains will be assessed.

- Selection bias: Bias due to inadequate randomisation method or allocation concealment method.
- Performance bias: Bias due to trial participants and personnel being aware of treatment allocation.
- Detection bias: Bias due to outcome assessors being aware of treatment allocation.
- Attrition bias: Bias due to the amount of missing data in a trial, differential missing data between trial arms, or inadequate methods of handling missing data.
- Reporting bias: Bias due to selective outcome reporting.
- Other sources of bias:
  a. Baseline imbalance: Bias due to imbalance in patient characteristics which are strongly related to treatment outcomes.
  b. Contamination bias: Bias due to participants randomised to one group receiving the protocol of a different group of the trial.
  c. Null bias: Bias due to incomplete implementation of treatment group protocol.
  d. Recruitment bias (cluster trials): Bias due to individuals being recruited after clusters are randomised.
  e. Incorrect analysis (cluster trials): Bias due to the analysis not taking the clustering into account.

Items will be rated for risk of bias as “Low risk”, “Unclear risk”, or “High risk” following the guidance in the Cochrane Risk of Bias Tool (Higgins et al., 2011). Performance, detection and attrition bias will be rated for each outcome extracted from a study.

We will rate the overall risk of bias for each outcome within a study using the following key domains: selection bias, detection bias and attrition bias. The overall risk of bias will be rated as “Low risk” if all key domains are rated as “Low risk”, “Unclear risk” if at least one key domain is rated as “Unclear risk” and none are rated as “High risk”, and “High risk” if at least one key domain is rated as “High risk”.

#### 3.7.3 Measures of treatment effect

##### Relative treatment effects

We will evaluate the same effect measures for both the pairwise and network meta‐analyses. For depressive symptom score, a continuous outcome, we anticipate that individual studies will use different measures and therefore we will estimate the effect using Hedges' g standardised mean difference. For acceptability, a dichotomous outcome, we will estimate the odds ratio (OR). We will report the summary effects and 95% confidence intervals (CIs) for each pair of treatments.

##### Relative treatment ranking

For each outcome, we will also estimate the probabilities for all treatments attaining each possible rank. This information will be used to develop a hierarchy of rankings using the surface under the cumulative ranking curve (SUCRA; Salanti et al., 2011). This approach to ranking accounts for the uncertainty in the treatment effects. A SUCRA value of 0% indicates the treatment is among the least effective of the treatments while a value of 100% indicates it is among the most effective.

#### 3.7.4 Unit of analysis issues

##### Cluster randomised trials

We will include cluster‐randomised trials in the analyses along with individually randomised trials. Where necessary, we will adjust standard errors using the methods described in the Cochrane Handbook (Higgins et al., 2011) using an estimate of the intracluster correlation coefficient derived from the trial if provided, from a similar trial, or from a study of a similar population.

We will also acknowledge heterogeneity in the randomisation unit and conduct a sensitivity analysis to investigate the effects of the randomisation unit.

##### Cross‐over trials

Cross‐over RCTs will be included only if they provide data at the end of the first stage. Data from the second stage, after crossover, will not be included.

##### Multiarm trial

As mentioned above, when there are two variations of the same mode of delivering of CBT along with a third relevant group, the two CBT groups will be combined. For continuous outcomes, this will be done by using the formulae provided in table 7.7a in the Cochrane Handbook (Higgins et al., 2011). For dichotomous outcomes, sample sizes and number of participants with outcome across the groups will be summed.

#### 3.7.5 Dealing with missing data

In case of missing information, the author(s) of the original study will be contacted. We will document correspondence with study authors.

We will prioritise analyses using intention‐to‐treat results. In cases where study authors used imputation to account for missing data within a study, we will prioritise the results based on multiple imputation, but will consider other imputation methods (e.g., last observation carried forward). Otherwise, we will use the available case analysis.

We will record the attrition rate and evaluate the risk of bias due to attrition bias.

#### 3.7.6 Assessment of heterogeneity

##### Assessment of clinical and methodological heterogeneity within comparisons

We will assess clinical and methodological heterogeneity by examining the distribution of extracted study, participant and intervention characteristics (described above) within each direct comparison.

##### Assessment of transitivity across treatment comparisons

We will assess the assumption of transitivity by comparing the distribution of the potential effect modifiers across the different pairwise comparisons. We will assess whether interventions are delivered similarly in trials with inactive control groups and trials with active controls; for example, whether one‐on‐one therapist‐led CBT is delivered similarly in trials comparing it to no intervention and trials comparing it to standardised self‐help.

#### 3.7.7 Assessment of reporting biases

We will aim to minimise the potential impact of reporting biases by conducting a comprehensive search for eligible studies and by being alert to duplication of data. We will use comparison‐adjusted funnel plots to explore publication bias and the possibility of small‐study effects across the network (Chaimani & Salanti, 2015). In order for the results of comparison‐adjusted funnel plots to be meaningful, the treatment comparisons need to be ordered consistently based on the anticipated direction of the small‐study effects. Therefore, anticipating that active treatments will be favoured, we will focus on active treatment versus inactive control comparisons. We will generate comparison‐adjusted forest plots using the netfunnel command in Stata 13® (Chaimani & Salanti, 2015).

#### 3.7.8 Data synthesis

##### Methods for direct treatment comparisons

A pairwise meta‐analysis will be conducted for each pair of interventions (or controls) where there are two or more head‐to‐head trials. As we anticipate that different trials within a comparison will not be estimating the same effect, we will use random effects models. We will perform pairwise meta‐analyses using the metan command in Stata 13® (Harris et al., 2008).

##### Methods for indirect and network comparisons

We will conduct network meta‐analyses using random effects models. These analyses will follow the multivariate metaregression approach accounting for correlations within multiarm trials (Lu & Ades, 2006; White, 2011; White, Barrett, Jackson, & Higgins, 2012). For the purpose of the analysis, we will set the most commonly used intervention (or control) among identified trials as the reference. We will use the “network” suite of commands in Stata 13® to conduct the network meta‐analyses (White, 2015).

##### Assessment of statistical heterogeneity

###### Assumptions when estimating the heterogeneity

We will conduct pairwise meta‐analyses assuming comparison‐specific heterogeneity (i.e., each direct comparison has a separate heterogeneity estimate). For network meta‐analyses, we will assume a common heterogeneity across comparisons.

###### Measures and tests for heterogeneity

Statistical heterogeneity within pairwise comparisons will be assessed through *χ* ^2^ tests and I^2^. We will consider the following thresholds when interpreting I^2^: 0–40% might not be important; 30–60% may represent moderate heterogeneity; 50–90% may represent substantial heterogeneity; and 75–100% represents considerable heterogeneity (Deeks, Higgins, & Altman, 2011). We will also consider the magnitude and the direction of the effects in our assessment of I^2^. To assess heterogeneity across the entire network, we will evaluate the magnitude of τ^2^ and compare it with the empirical distribution (Rhodes, Turner, & Higgins, 2015; Turner, Davey, Clarke, Thompson, & Higgins, 2012).

###### Assessment of statistical inconsistency

We will evaluate inconsistency using a combination of local and global approaches. If we detect inconsistency, we will carefully re‐evaluate the set of studies indicated by the tests which may be the source of inconsistency.

###### Local approaches for evaluating inconsistency

We will evaluate inconsistency locally using the loop‐specific approach and the node‐splitting approach.

The loop‐specific approach involves examining each closed loop of at least three treatments to determine the agreement between direct and indirect evidence (Higgins et al., 2012). The difference between the direct and indirect estimate is represented by the inconsistency factor and its 95% CI and if the 95% CI is not compatible with 0, it indicates the presence of potential inconsistency. We will implement the loop‐specific approach using if plot in Stata 13® (Chaimani & Salanti, 2015).

The node‐splitting approach involves examining each pair of treatments individually to compare the direct and indirect estimates (Dias, Welton, Caldwell, & Ades, 2010). Significant differences indicating potential inconsistency are detected using a *z* test. We will implement the node‐splitting approach using the network sidesplit command in Stata 13® (White, 2015).

###### Global approaches for evaluating inconsistency

We will evaluate inconsistency in the entire network simultaneously using a design‐by‐treatment interaction model. This model adds terms to represent disagreement between direct and indirect evidence as well as differences by trial design (e.g., two‐arm versus three‐arm trials; Higgins et al., 2012). A Wald test is used to assess potential inconsistency. We will fit inconsistency models using the “network” suite in Stata 13® (White, 2015).

##### Summary of findings

The main summary of findings table will be based on GRADE recommendations. The table will include the quality of evidence, effect size (against a control group), sample size and direct or indirect evidence, with further details summarised in a narrative and additional tables (capturing the intervention details and study details). The adaptation of GRADE to network meta‐analysis will be implemented using the CINeMA web application (http://cinema.ispm.ch/). We will also assess the certainty of treatment rankings based on the Salanti framework (Salanti, Giovane, Chaimani, Caldwell, & Higgins, 2014). The geometry of the network will be described according to the PRISMA guidelines (Hutton et al., 2015). We will also present the findings including effect size (against a control group), confidence intervals, SUCRA rankings and quality of evidence using a bar graph. We will summarise the relative effectiveness of all interventions against each other in a matrix.

#### 3.7.9 Subgroup analysis and investigation of heterogeneity

One potential effect modifier is participant age (Curry et al., 2006). We will conduct some exploratory subgroup analyses to investigate the effect of participant age on the primary outcome. Subgroup analyses will be conducted to investigate differences by splitting studies according to mean age of participants as follows: 10 to 13 years; 14 to 15 years; ≥16 years. These subgroups are based on a study that found differences between age subgroups on response to treatment for depression in adolescents (Curry et al., 2006). We will examine the differences in the results of these subgroup analyses qualitatively and if there are substantial differences, we may re‐evaluate transitivity. For example, if the analysis of the youngest participants has substantially different results from that of the older groups, we may exclude these studies from the primary analysis.

#### 3.7.10 Sensitivity analysis

As mentioned above, if we are able to identify differences between interventions in terms of components such as behavioural activation, problem solving, social skills training or relaxation techniques, we will test whether these differences may confound the estimated difference between delivery modes using sensitivity analyses.

In addition, all placebo control conditions will be grouped together in the same node in the network, but a sensitivity analysis separating therapist‐led placebos, self‐help placebos and pill placebos will be carried out if these different types of placebos are present in the included studies.

We will also conduct a sensitivity analysis to exclude studies where symptoms of depression were established using an unvalidated measure or unclear method.

We will also conduct a sensitivity analysis to exclude studies where we have coded the intervention as customised to examine whether customisation of interventions may confound the estimated difference between delivery modes.

Finally, we will conduct a sensitivity analysis to investigate the effects of the randomisation unit if cluster‐randomised and individually randomised trials are identified and included in the analysis.

Sensitivity analyses will be conducted for the primary outcome at the postintervention time point.

## CONFLICT OF INTERESTS

None of the authors have been involved in the development of any relevant interventions or primary research nor have they published a prior review on the topic.

## AUTHOR CONTRIBUTIONS

G. B. and L. T. will provide the content of the review that is pertinent to adolescent depression and interventions delivered via technology. S. S. will provide the methodological content, in consultation with G. B. and B. R. G. B. and N. A. will review and edit all content.

G. B., S. S. and B. R. will design the methodology for the review, with suggestions and input from Nick Axford. S. S. has worked on various reviews of evidence‐based programmes, including a Cochrane‐style systematic review. B. R. has coauthored several network meta‐analysis and has written guidance for clinicians in conducting and interpreting network meta‐analyses. G. B. has previously coauthored two Cochrane systematic reviews involving meta‐analysis of interventions for behaviour problems in children and has taken a short course in Network Meta‐Analysis at the University of Oxford. N. A. has a wealth of experience in comprehensive rapid evidence reviews and led the Dartington Social Research Unit's rigorous review process for the Blueprints for Healthy Youth Development database (assessing the quality of experimental evaluation).

B. R. will perform the statistical analyses, has experience in conducting network meta‐analysis and is familiar with multiple programs for network meta‐analysis, including Stata and WinBUGS.

Screening, data extraction and assessment of the risk of bias will be conducted by S. S., L. W. and B. R. with input from G. B. if required. S. S. has extensive experience in searching databases and conducting reviews through her work on the Blueprints for Healthy Youth Development database and a related European Commission project, and other rapid evidence reviews. L. W. has experience screening and coding studies for meta‐analysis for the Investing in Children project, as well as for a rapid evidence review to update evidence for the Healthy Child Programme for Public Health England. B. R. has extensive experience in conducting systematic reviews and network meta‐analyses with the Cochrane Eyes and Vision Group and the Evidence‐based Practice Center at the ECRI Institute. All have experience in coding and critically appraising studies.

**Additional tables**

1 Examples of measures of depression

**Table:** 

Measure	Score cut point
Beck Depression Inventory Second Edition	≥14 (http://academicdepartments.musc.edu/family_medicine/rcmar/beck.htm)
Centre for Epidemiologic Studies Depression Scale Revised	≥16 (http://cesd‐r.com/cesdr/)
Children's Depression Inventory	≥16 (Ivarsson, Svalander, and Litlere (2006); Roelofs et al., 2010)
Children's Depression Rating Scale‐Revised	≥30 (based on author correspondence)
Children's Depression Scale	≥135 (Tisher, Lang‐Takac, & Lang, 1992)
Hamilton Depression Rating Scale	≥8 (Hamilton 1960; Sharp 2015) *Dependent on the version of the HDRS used in the study
Mood and Feelings Questionnaire	≥27 (based on author correspondence)
	≥5 on the short version (SMFQ; Thapar & McGuffin, 1998)
Reynolds Adolescent Depression Scale (RCDS)	*t* score of 61 equivalent to a raw score of 76 (Reynolds 2004)
Patient Health Questionnaire–9	≥5 (Kroenke & Spitzer, 2002)
Patient Health Questionnaire for Adolescents (PHQ‐A, also called the Severity Measure for Depression)	≥5 (Johnson, Harris, Spitzer, & Williams, 2002)

**Sources of support**

**Internal sources**

The time of Nick Axford is supported by the National Institute for Health Research Applied Research Collaboration South West Peninsula. The views expressed are those of the author(s) and not necessarily those of the NHS, the NIHR or the Department of Health and Social Care.

**External sources**

- Jacobs Foundation, Switzerland

This protocol is funded by the Jacobs Foundation through the Better Evidence for Children and Youth programme. No other funding has been sought.
